# Adipocytes as lipid sensors of oleic acid transport through a functional Caco-2/HT29-MTX intestinal barrier

**DOI:** 10.1080/21623945.2019.1580842

**Published:** 2019-03-23

**Authors:** Emmanuelle Berger, Alain Géloën

**Affiliations:** CarMeN Laboratory, INRA UMR1397, INSERM U1060, INSA-Lyon, IMBL, Université Lyon 1, Lyon, France

**Keywords:** Adipocyte, hepatocyte, enterocyte, oleic acid, lipogenesis

## Abstract

Adipose tissue function in the regulation of lipemia is highly dependent on intestinal absorption of nutrients. Therefore the aim of the present study was the development and validation of an *in vitro* multiculture model allowing to measure intestinal absorption using adipocytes as lipid sensors. We previously described (1) novel methods to study oleic acid induction of adipogenesis and lipogenesis and (2) a functional reconstituted intestinal barrier using human cell lines Caco-2/HT29-MTX (9:1). In the present study we develop a co-culture model with either adipocytes or hepatocytes as sensors for intestinal lipid absorption. This model was validated using oleic acid (OA) pre-absorbed onto the intestinal barrier. Optimized experimental conditions were obtained with partially differentiated 3T3L1-MBX adipocytes sensing up to 5 μM OA in solution or 40 μM OA pre-absorbed by Caco2/HT29-MTX intestinal barriers. Metabolism including glycemia and insulinemia greatly influenced the ability to  TG accumulation in adipocytes. By comparison AML12 hepatocytes found less sensitive to OA (up to 1 μM). The present study demonstrates a much better functionality for fatty acid uptake and release in Caco2/HT29-MTX *versus* Caco-2 intestinal barriers. Taken together these results open new opportunities to study *in vitro* lipid transfer between intestinal barriers and either adipocytes or hepatocytes.

**Abbreviations:** BSA: Bovine serum albumin; CIDEs: Cell Death Inducing DFFA Like Effectors; DMEM, Dulbecco’s Modified Eagle’s Medium; FABPs: Fatty Acid Binding Proteins; FAT/CD36: Fatty acid translocase; FCS: Fetal calf serum; GLP2: Glucagon-like peptide-2; NAFLD: Nonalcoholic fatty liver disease; OA: oleic acid; PBS: Phosphate buffer saline; PPARs: Peroxisome-Proliferator Activated Receptors; RTCA: realtime cell analysis; TG: triglyceride

## Graphical Abstract

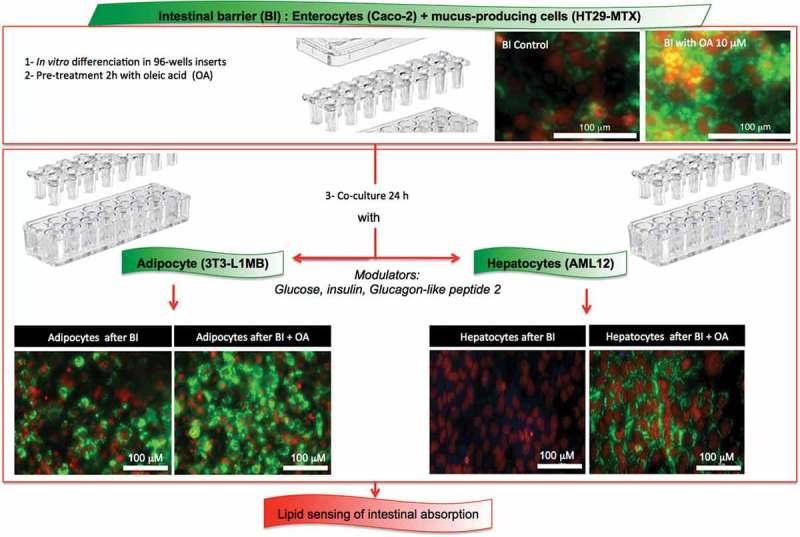

## 1. Introduction

Lipids are essential to insure life as a source of essential fatty acids which cannot be synthesized by the organisms [1]. Few cell types in homeotherms are able to store lipids as triglycerides (TG), it is the case for adipose cells, which main function is to maintain lipid homeostasis through lipid storage, and for hepatocytes, although fat accumulation in these cells is the signature of metabolic alterations [2–4]. Chronic liver diseases represent an important, and underestimated, global public health problem [5]. Worldwide estimations show that 844 million people have chronic liver diseases, with a mortality rate of 2 million deaths per year [6]. Nonalcoholic fatty liver disease (NAFLD) is highly prevalent in rich and developing countries with an incidence close to 25% of the population in Europe [7,8]. In hepatocytes TG accumulation is induced by increased influx of fatty acids from the diet, *de novo* lipogenesis, and free fatty acids (FFAs) liberated from the adipose tissue and its excess is largely involved in NAFLD [9]. The development of a simple and robust cellular model to study lipogenesis and the consequences of intracellular TG accumulation would accelerate the discovery of the mechanisms involved in its dysregulation. We have recently published a method to monitor lipogenesis based on the real-time measure of the impedance of adipocytes [10]. Such a real-time and noninvasive method shows strong reproducibility and high sensitivity. Besides, we have recently characterized an *in vitro* model of intestinal barrier from a co-culture of Caco-2 plus 10% HT29-MTX cell lines [11]. In that study we showed that oleic acid rescued healthy enterocyte phenotype in the co-culture of these two colon cancer cell lines. The aim of the present study was, by combining the reconstructed intestinal barrier and the measure of lipogenesis, to develop an *in vitro* model of nutrient absorptive system. Indeed using that combination, the efficiency of absorption of the reconstructed intestinal barrier will be evaluated by the real-time measurement of oleic acid-induced lipogenesis in both adipose and hepatic target cells. Besides demonstrating the feasibility of such a system we have compared the answers of the *in vitro* differentiated cell lines 3T3L1-MBX adipocytes and AML12 hepatocytes to oleic acid pre-absorbed on the apical site of the reconstructed intestinal barrier.

## 2. Results

### 2.1. Effect of differentiation time, glucose and insulin on lipogenesis

**Figure 1.**
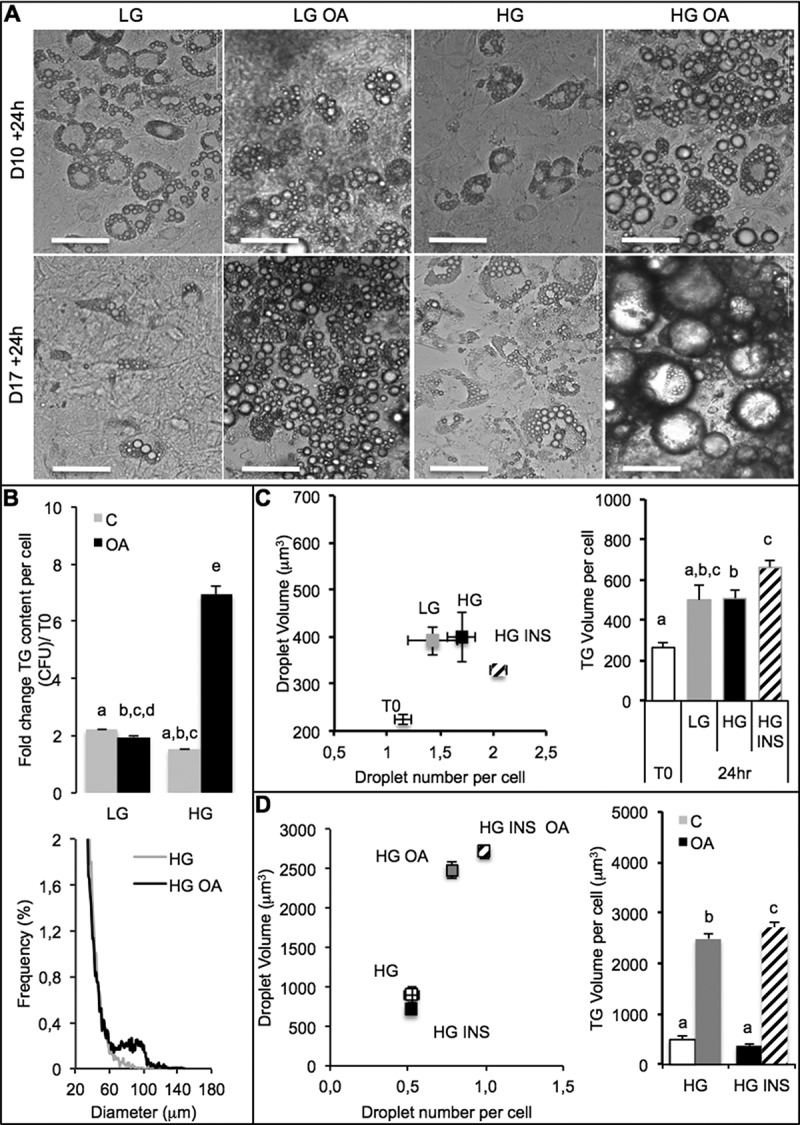
Culture media and oleic acid influence 3T3L1-MBX adipocytes. 3T3L-MBX fibroblasts were differentiated *in vitro* then treated with culture media containing glucose at either 1 g/L (low LG), 4.5 g/L (high HG), insulin (INS 0,01 mU/mL) and/or oleic acid (OA 10 µM) *versus* vehicle (Control C) during 24 h. (a) Bright field imaging of poorly (Day 10 post-differentiation) *versus* highly differentiated (D17) adipocytes treated during 24 h. (Scale bars = 50 µm). (b) Triglycerides (TG) content analysis by fluorescent quantification (CFU) of AdipoRed normalized to Hoechst 33258 (nuclei, i.e. cell number, upper) and size distribution of cells treated in HG (lower) without or with oleic acid (10 µM). (c) Lipid droplet size and number analyzed by AdipoRed counts normalized to those of Hoechst (X4 magnification on at least 5 fields) in several medias. (d) Effect of treatment with OA (10 µM) in HG media. Data are presented for representative experiments by mean values ± SEM (at least 5 replicates). Anova variance analyses are reported as letters.

Prior co-culture analyses, the response of adipocytes to oleic acid (OA) was studied under different experimental conditions. Time of exposure should also be taken into account. Preliminary studies indicated that intestinal barriers could be loaded onto both adipocytes and hepatocytes during 24 h without significant toxic nor morphological modification (not shown). Moreover the stage of differentiation of adipose cells, the concentration of glucose and the presence of insulin may highly modulate lipogenesis. Indeed, lipogenesis was more important in response to the same concentration of OA (10 µM) when fat cells were longer differentiated (10 days *versus* 17 days, Figure 1(a)). High glucose concentration increased TG accumulation induced by OA, compared with low glucose concentration (Figure 1(b)). In living organisms high glucose concentration induces insulin release, for that reason we tested the effect of insulin on lipogenesis. High glucose concentration compared with low glucose concentration had slight effect on lipogenesis. Insulin in presence of high glucose increased TG content within adipose cells (Figure 1(c)). When OA and insulin were both present, not only the number but also the size of lipid droplets within adipose cells increased resulting in a significant increase of TG content (Figure 1(d)).

### 2.2 Dose-dependent responses to OA of partially differentiated adipocytes

**Figure 2.**
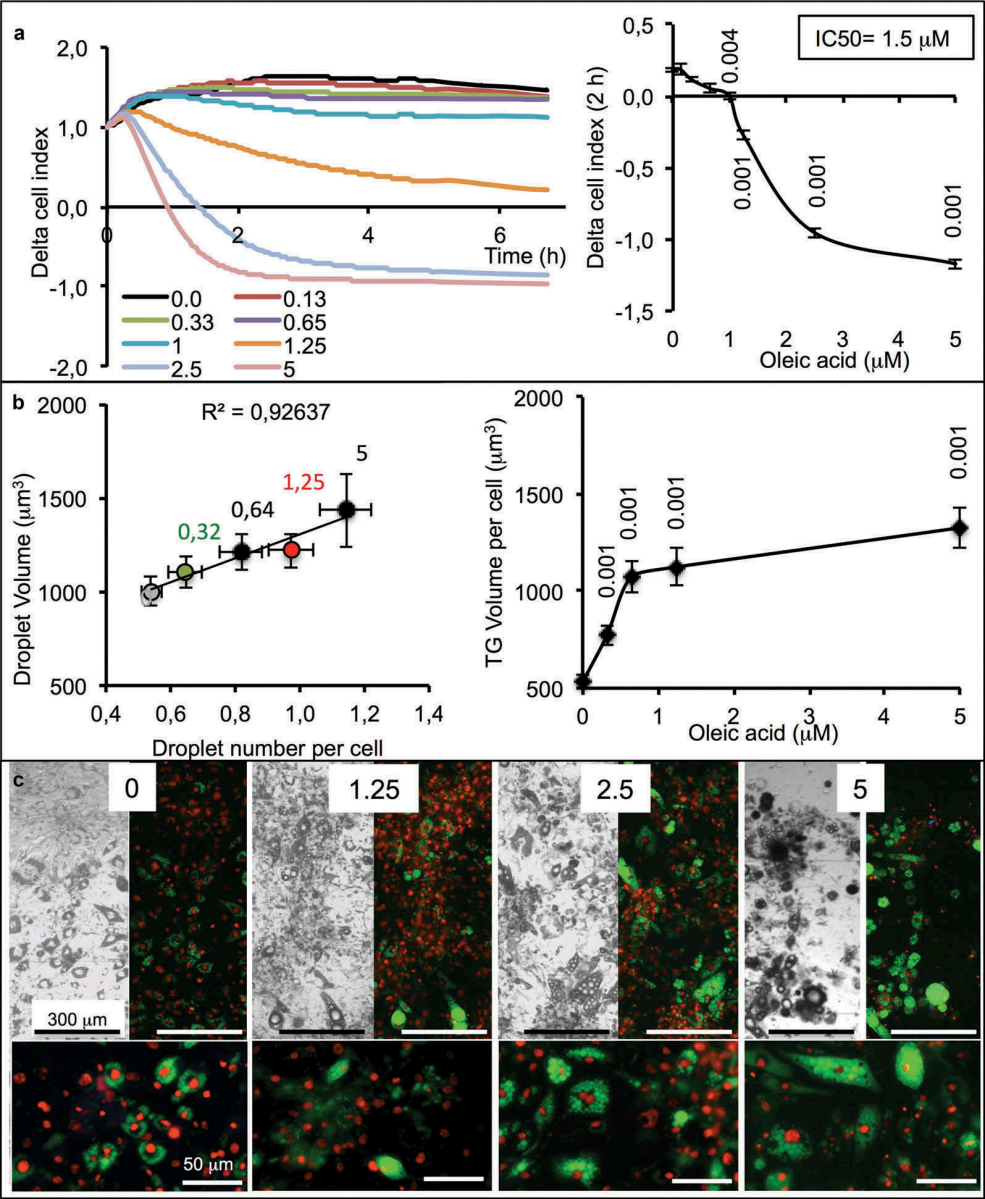
Dose-response of partially differentiated 3T3L1-MBX adipocytes (D5) to oleic acid (OA) during 24 h (High glucose). (a) RTCA analysis of dose-dependent response to OA is highly sensitive and reveals cell adhesion force reduction due to TG accumulation within few hours. IC50 was of 1.5 µM and allowed TG accumulation up to 5 µM (B). Higher concentrations of OA increased droplet volumes (i.e. TG accumulation) and number (until 5 µM), thus increasing intracellular TG content in a dose-dependent manner to OA concentration. (c) Bright field and fluorescent merged images of cell cultures (objective x4, upper panels) nuclei were labeled with Hoechst 33258 (red) and lipid droplets with AdipoRed (green), lower line (objective x20, lower panels). Data are presented for representative experiments as mean values ± SEM (at least 8 replicates) with significant Student t-test p-values (p < 0.05) *versus* controls.

To be used for lipid sensing, adipocytes must be highly sensitive to small OA amount uptake, thus partially differentiated. When incubated on xCELLigence in presence of increasing concentrations of OA, the cell index slightly decreased compared with control in response to the lowest OA concentrations and then decreased more rapidly from 1 µM to 5 µM OA, resulting in an IC50 = 1.5 µM (Figure 2(a)).

The cell index is influenced by cell number (i.e. proliferation *vs*. death), cell size (i.e. morphological changes) and adhesion cell force (i.e. lipid content). Cells do not proliferate after the induction of adipocyte differentiation and low concentrations of OA have no effect on cell survival, so the cell index should remain constant except when TG accumulation decreases the adhesion strength of cell, consequently reducing the cell index values. One limitation of xCELLigence is that when the cell index is close to zero, it is then not possible to further measure the lipogenesis. An alternative is then to switch to another method, such as cell fluorescence imaging using microscopy. In that case, it was then possible to measure the size and number of lipid droplets. The results show that a low concentration of OA was sufficient to increase the number of lipid droplets per cell and only in response to the highest OA concentration an increase of lipid droplet volumes. On the all, lipid contents increased in response to increased OA concentrations until 5 µM (Figure 2(b)). Visual controls of adipose cell show that all the cells accumulated TG. Although the total amount of TG per cell is variable, all the cells accumulate TG according to OA concentrations (Figure 2(c)).

### 2.3. Effect of the nature of the intestinal barrier on TG storage in adipose cells

**Figure 3.**
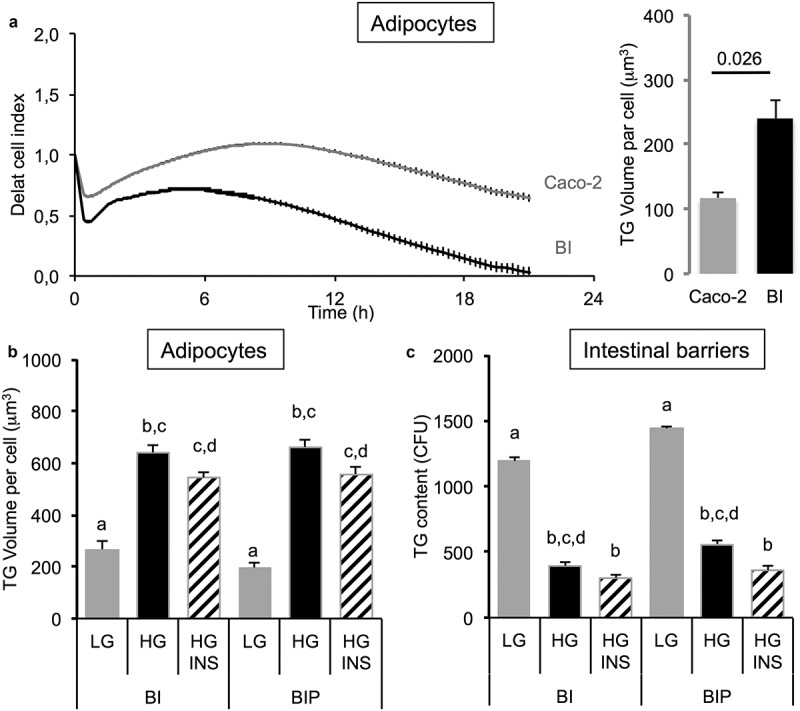
Influence of co-culture media in TG storage by 3T3L1-MBX adipocytes co-cultured with intestinal barriers. (a) Real-time analysis of adipocytes co-cultured with intestinal barriers Caco-2 or Caco-2/HT29-MTX (BI, left panel) and TG content after 24 h (right panel). TG content in (B) adipocytes and (C) intestinal barriers (BI) *versus* primed (BIP) 24 h after co-culture in either low (LG) or high (HG) glucose media with insulin (INS 0.01 mU/mL). Data are presented as mean values ± SEM (n = 8) with significant Student t-test p-values (p < 0.05) or Anova variances (letters).

The single presence of intestinal barrier induced a decrease of cell index in adipose cells attesting an increased intracellular accumulation of TG (Figure 3(a)). This was observed when the intestinal barrier was made of Caco-2/HT29-MTX (BI) but not in the presence of Caco-2 cells alone. Such a result was confirmed by the measure of TG content in adipose cells. It is noticeable that the simple presence of the intestinal barrier, without any addition of OA, resulted in increased lipid content in adipose cells. Besides the co-culture, the presence of the intestinal barrier, the concentration of glucose and the presence of insulin impacted the TG volume within adipose cells. Indeed, high glucose concentration increased TG volume compared to low glucose concentration (Figure 3(b)). Insulin in presence of high glucose exerted little effect compared with high glucose alone when the intestinal barrier was not primed (BI). The preliminary priming of intestinal barrier with OA (i.e. BIP, pre-treated with OA 10 μM during 2 h then maintained in culture media during 24 h in order to induce intestinal barrier maturation), had little effect on lipid content in adipose cells (Figure 3(b)). Lipid contents within the intestinal barrier perfectly matched with the lipid contents measured within adipose cells. Low glucose concentration resulted in a high lipid content in the intestinal barrier and low in adipose cells while it was the opposite with high concentration of glucose (Figure 3(b)).

**Figure 4.**
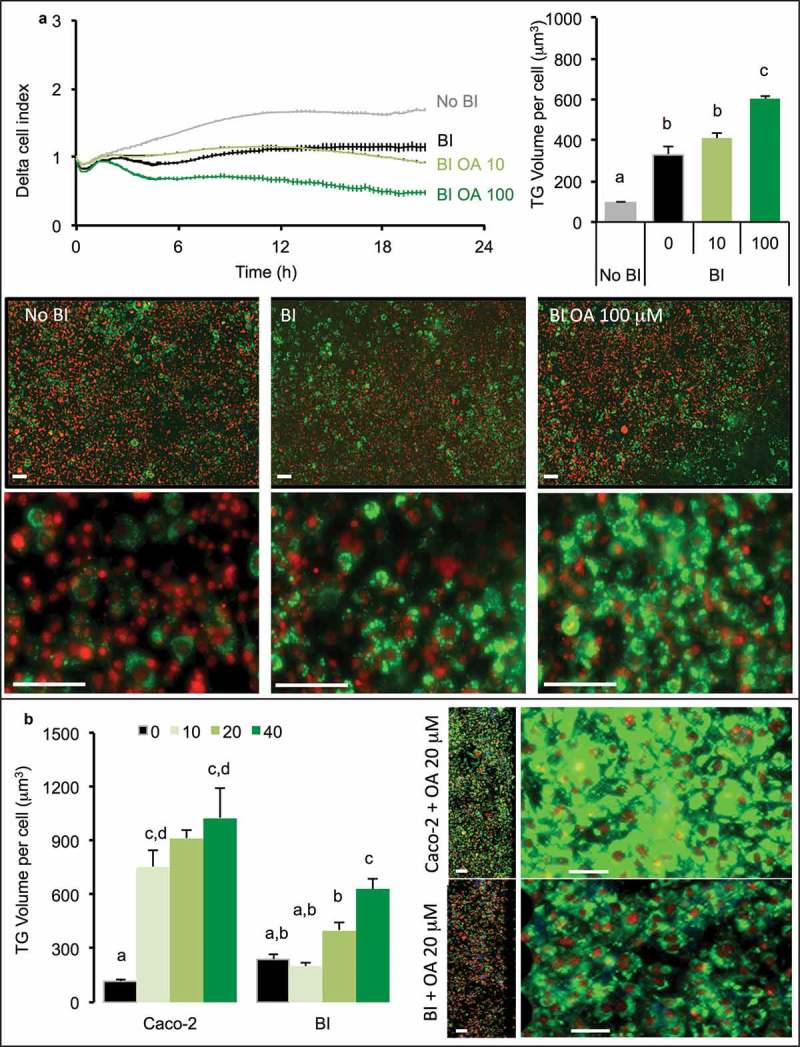
3T3L1-MBX adipocytes sensing of oleic acid (OA) pre-absorbed by intestinal barriers Caco-2 and Caco-2/HT29-MTX (BI). (a) Real-time analysis of adipocytes (left upper panel) co-cultured with BI pre-treated 2h with OA 10 or 100 µM or control media (co-culture in HG media) reveals significant TG accumulation after AdipoRed lipid droplet volume analysis (right upper panel). (b) Comparative sensing in adipocytes 24 h after co-culture with either Caco-2 or Caco-2/HT29-MTX (BI) pre-treated with several doses of OA (μM). Data are presented as mean values ± SEM (at least 5 replicates) with Anova variances (letters). Corresponding merged AdipoRed (green) and Hoechst 33258 (red) images were acquired at either x4 or x20 magnification using imaging parameters normalized to controls.

Oleic acid was applied at either 10 or 100 μM during 2 h on the apical side of intestinal barrier (Caco-2/HT29-MTX) then removed before loading onto adipose cells, the intracellular storage of TG increased dose-dependently in adipose cells (Figure 4(a)). That result was observed through the measure of cell index which decreased, by measuring TG volume within adipose cells which increased and was confirmed by microscopy. The comparison of intestinal barriers made of Caco-2 only or co-culture (Caco-2/HT29-MTX) showed different results (Figure 4(b)). Indeed, after pre-treatment with OA, Caco-2 intestinal barriers induced a rapid increase of TG content within adipose cells and a very small dose-dependence effect of the OA concentrations was observed. On the contrary, an intestinal barrier containing HT29-MTX cells induced a more limited TG uptake within adipose cells but a better dose-dependency, confirmed by microscopy images.

### 2.4. Enterocytes accumulate TG when co-cultured with Caco-2/HT29-MTX intestinal barriers pre-treated with oleic acid

**Figure 5.**
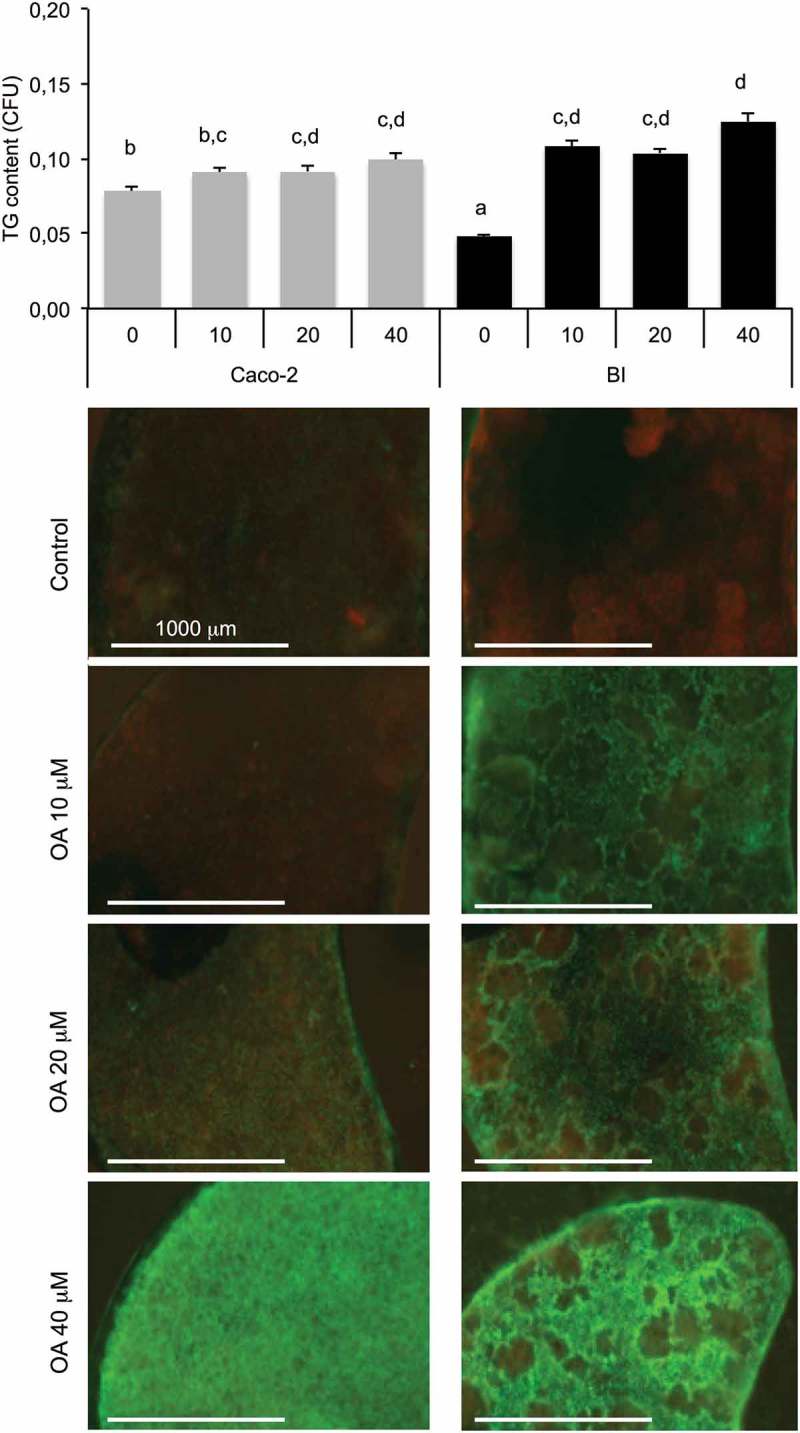
Oleic acid (OA)-induced TG retention in Caco-2 or in Caco-2/HT29-MTX (BI) 24 h post-treatment. Caco-2 and BI, pre-treated with OA 10 µM during 2 h were then maintained in culture media (HG) during 24 h before analysis of TG content using AdipoRed labeling. Data are presented as mean values ± SEM (8 replicates) with Anova variances (letters). Corresponding merged AdipoRed (green) and Hoechst 3358 (red) images were obtained at identical acquisition parameters using objective x4.

In the following experiments, we questioned whether the OA uptake and storage could change depending on the nature of the intestinal barrier. We then measured the uptake of OA by intestinal barrier according to their composition and their treatment. The Caco-2/HT29-MTX barrier showed a slight accumulation from 0 to 20 µM OA, and increased in response to 40 µM. The intestinal barrier composed of Caco-2cells showed a low lipid content under basal condition. Pre-exposure to OA increased its lipid content without significant dose-response effect (Figure 5).

### 2.5. Lipid sensing by adipocytes exposed to reconstructed intestinal barrier (Caco-2/HT29-MTX)

**Figure 6.**
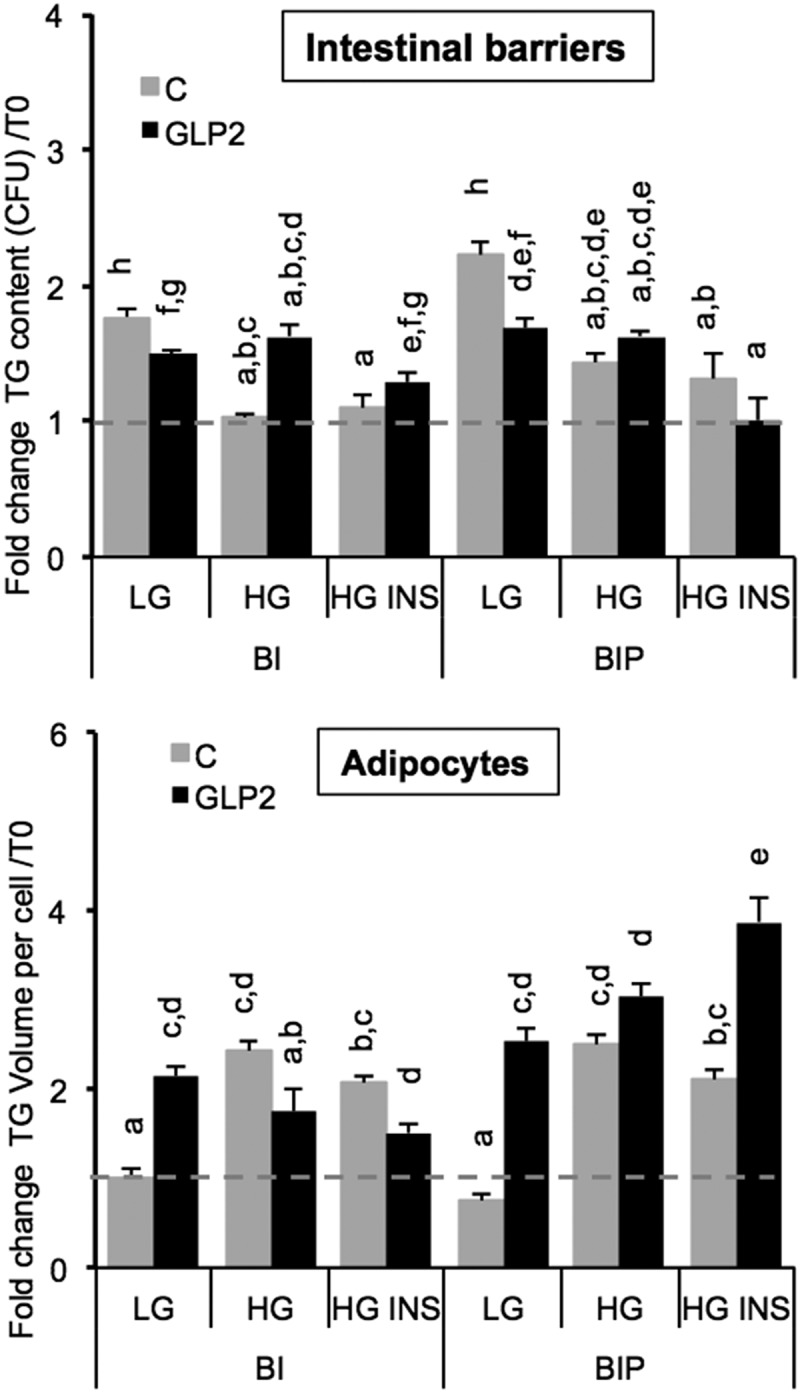
Lipid content in intestinal barriers pre-treated or not with GLP2 and in 3T3L1-MBX adipocytes. Intestinal barriers Caco-2/HT29-MTX without (BI) or with priming (BIP) were pre-treated with GLP2, 125 ng/mL during 2 h then co-cultured with 3T3L1-MBX adipocytes during 24 h in either low (LG) or high glucose (HG) containing insulin (INS 0.01 mU/mL). Contents of triglycerides (TG) were monitored using AdipoRed labeling and normalization to time of starting co-cultures (T0). Data are presented as mean values ± SEM (8 replicates) with Anova variance (letters).

We observed in previous experiments that intestinal barriers accumulated lipids after exposure to OA in a dose-dependent manner [11] and 24 h later, high TG contents were detected in BI. Such retention in BI suggests regulatory mechanisms controlling TG release by BI. Since the role of GLP2 in chylomicron excretion has been well *stated*, we tested its role under different conditions such as low or high glucose concentrations and presence of insulin, on co-cultured intestinal barriers in the presence of adipose cells [12]. Intestinal barriers were pre-treated with GLP2 125 ng/mL (in low glucose DMEM media) during 2 h before being placed in presence of adipose cells. Results showed that GLP2 decreased lipid content significantly in primed but not significantly in non-primed intestinal barriers (Figure 6) in low glucose co-culture media. GLP2 increased lipid content in non-primed intestinal barrier in presence of high glucose concentration, that effect was not present when the intestinal barrier was primed. GLP2 had no effect on either primed or non-primed intestinal barriers in high glucose plus insulin.

On the whole, GLP2 increased lipid content in adipose cells, it is the case for non-primed intestinal barriers in low glucose and for primed intestinal barriers in low glucose and high glucose plus insulin. The condition in which GLP2 deceased lipid content in adipose cells is in presence of non-primed intestinal barriers in high glucose with or without insulin. The highest lipid content in adipose cells was observed after co-culture with primed barriers under high glucose plus insulin. Finally it can be concluded that adipocyte responses to GLP2 treatment in the three co-culture media were inversely correlated with those of intestinal barriers 24 h after co-culture. Moreover, the sensitivity measured in adipocytes was higher than that of intestinal barriers (fold changes in a range of 6 and 4, respectively).

### 2.6. Lipid sensing by hepatocytes exposed to reconstructed intestinal barriers

**Figure 7.**
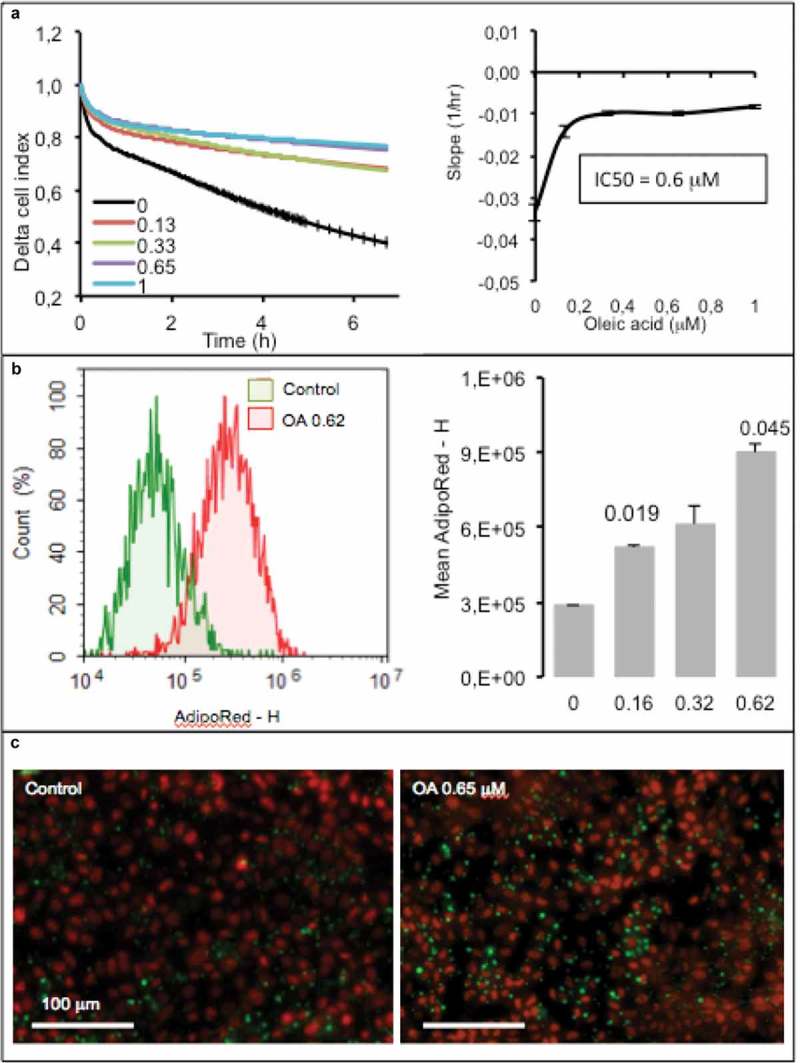
Dose-response of hepatocytes AML12 to oleic acid (OA) after 24 h. (a) Real-time analysis of OA uptake by enterocytes AML12 (left panel) and corresponding slopes and IC50 (right panel). Data are presented as mean values ± SEM (8 replicates). (b) OA-induced TG accumulation monitored by cytometry analysis of AdipoRed fluorescence. Mean AdipoRed-H was analyzed on living cell populations in duplicates (right panel). (c) Merged images of AdipoRed (green) and Hoechst 33258 (red) acquired with identical parameters with objective x20. Significant Student t-test-p-values are reported for p < 0.05, compared to control.

In the following series of experiments, we wanted to test whether hepatocytes could be used for lipid sensing. For that purpose, AML12 cells were plated on xCELLigence  E-plates and exposed to increasing concentrations of OA. In control cells, the cell index reduction was induced by absence of growing factors without affecting cell survival. Unlike fat cells the cell index of hepatocytes increased in response to OA, in a dose-response manner, resulting in an IC50 of 0.6 µM (Figure 7(a)). Intracellular lipid monitored using flow cytometry with AdipoRed as a marker showed a perfect increase of the labeling in response to the OA and quantification of mean Adipored fluorescence intensity (Figure 7(b)). These results were confirmed by microscopy imaging (Figure 7(c)).

**Figure 8.**
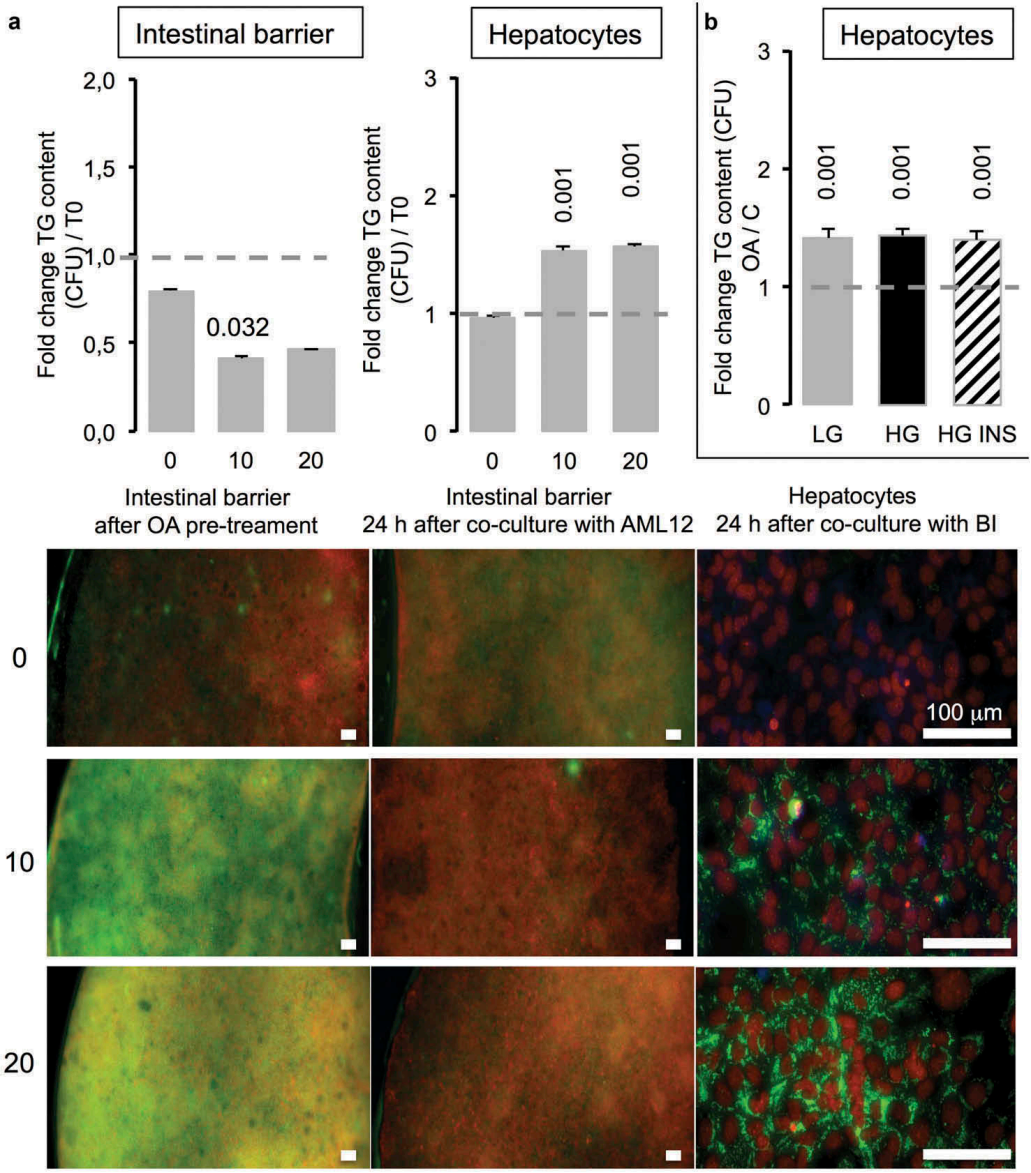
Caco-2/HT29-MTX intestinal barriers pre-treated with oleic acid (OA) in co-culture induce TG accumulation in AML12 hepatocytes. (**a)** Intestinal barriers were pre-treated with either 10 or 20 μM OA during 2 h then OA was removed by replacing media and intestinal barrier 96-wells inserts were loaded onto AML12 hepatocytes during 24 h in HG media. TG accumulation was assessed using AdipoRed fluorescent intensities normalized to time of pre-treated intestinal barrier before loading onto AML12 cells in either Caco-2/HT29-MTX (T0) or 24 h after co-culture (A) and AML12 cells. Corresponding images are presented as merged AdipoRed (green)/Hoechst 33258 (red) images at magnification x4 (96-wells inserts) or x20 (AML12). Dot line at 1 represents the value for control at T = 0. (b) AML12 sensing of intestinal barriers pre-treated with OA (10 µM) in co-culture media containing either low (LG) or high glucose (HG) with or without insulin (INS 0,05 mU/mL) after 24 h. Data are presented as mean values ± SEM (n = 8) with significant Student t-test p-values <0.05 compared to T0.

In the next step, co-cultured intestinal barriers have been exposed to 10 or 20 µM OA before being placed on the top of hepatocytes (T0). After 2 h, the TG content in green (AdipoRed) in intestinal barriers was significantly increased compared to the one of barriers not exposed to OA, although there was no difference between barrier exposed to 10 *versus* 20 µM OA (Figure 8(a), left images). Twenty four hours after co-culture with hepatocytes, the lipid content decreased in BI. Inversely, in hepatocytes the presence of TG was significantly increased even at the lowest OA concentration (10 µM). We observed no difference in lipid content of hepatocytes when the BI was loaded with OA at 10 or 20 μM although the TG content in intestinal barrier almost disappeared after 24 h (Figure 8).

## 3. Discussion

The present study establishes that it is possible to monitor lipid uptake and release through a reconstructed intestinal co-cultured barrier using a third cell type, either adipocytes or hepatocytes, as lipid sensor. Conditions of cell culture are of course critical for several aspects.

- The intestinal barrier made of Caco-2 cells was less functional than the co-culture Caco-2/HT29-MTX for OA uptake and storage in their capacity to control retention *versus* basal lipid release. Caco-2 alone exerted no effect on adipose cells while the co-culture itself provoked a reduction of cell index on adipose cells (Figure 3(a)) suggesting a reduced reactivity of Caco-2 barriers compared with the co-culture. In a previous study, we found that both intestinal barriers accumulate OA in a dose-dependent manner [11]. However 24 h after OA pre-treatment (priming), the remaining TG content was not higher than 1.3 fold in Caco-2 in comparison to untreated control, although 2 to 2.3 in Caco-2/HT29-MTX cells (Figure 3(a)) and inversely, Caco-2/HT29-MTX induced an increased of TG, 2 fold higher than Caco-2 in adipose cells. This effect was independent of the presence of adipocytes, since similar effect was observed in both intestinal barriers 24 h co-cultured with hepatocytes or in absence of sensor cells (not shown). Pre-exposure to increasing OA concentrations produced only a limited retention of lipids inside the Caco-2 barrier. After exposure to increasing OA concentrations, the lipid content in Caco-2 barrier was lower than in the co-culture. The 24 h storage capacity of OA was much bigger in the co-culture than in Caco-2 cells alone mainly because the basal value was much lower in the co-culture. Such a difference suggests different regulations of lipid uptake and release in both models. A number of fatty acid transporters have been characterized and extensively studied, like Fatty Acid Binding Proteins (FABPs) or Fatty acid translocase FAT/CD36 which are involved in the control of TG retention in enterocytes [13]. **I**n a previous study [11], several signaling pathways were commonly identified in cancers as potentially dysregulated in Caco-2 barriers, including that of fatty acids. We also evidenced a transcriptional down-regulation of FABPs as well as several lipid-droplet associated proteins in Caco-2/HT29-MTX *versus* Caco-2 intestinal barriers. Such differences may be related to higher retention capacities in Caco-2/HT29-MTX *versus* Caco-2 barriers. As a matter of fact, the co-culture appears a much better model to study lipid uptake and release than Caco-2 cells alone.

- Not only the nature but also the pre-treatment of the intestinal barrier is important. In a previous study we showed that the co-culture of Caco-2 plus mucus-producing cells HT29-MTX rescued the enterocyte phenotype from cancer cells, a phenomenon that was improved by the addition of OA [11]. In the present study, the priming of the intestinal barrier (i.e. OA pre-treatment during 24 h) resulted in an increased capacity of lipid uptake by adipose cells than in non-primed barriers, thus improving the functionality of the intestinal barrier after priming (Figure 6).

- xCELLigence is a highly sensitive method to measure lipogenesis within adipose cells in real-time experiments [10]. The cell index takes into account cell number, cell size and adhesion cell force. In the present case, adipocytes do not proliferate, their cell sizes increase because of lipid deposition. Although cell death of poorly differentiated cells exposed to oleic acid treatment cannot be excluded, the adhesion force decreases mainly because intra-cellular lipid content increases, and consequently reduces the adhesion surface and adhesion strength. Depending on the lipid concentration at which adipose cells are exposed, cell index reaches zero more or less rapidly without effect on cell survival [10]. In the present study, the utilization of xCELLigence required to use adipose cells that are not too advanced in differentiation, in order to keep a cell index high enough, indeed, adipose cells too advanced in differentiation would produce a cell index close to zero, with no or too little margin to study lipogenesis.

Nevertheless it is also possible to quantify lipid deposition in adipose cells using microscopy with the advantage that it makes possible to discriminate the number and the size of lipid droplets. Such a method gives then a better characterization of adipose cell differentiation, since differentiation efficiency may also differ from an experiment to another. The 3T3L1-MBX cell line can be fully differentiated (100% of cells with lipid droplets). During differentiation, lipid droplet size increases due to lipid accumulation. Fully differentiated cells become unilocular, thus resemble mature adipocytes. However such a maturation in culture requires additional treatment with fatty acids in complement to standard differentiation procedure (Figure 1). The sensitivity of adipose cells to lipid accumulation depends on cell size: more enlarged are the droplets, more concentrated fatty acid solutions should be applied to detect the differences [10]. In another way, the limit of fatty acid concentrations which could be applied onto the intestinal barriers (without toxic effect), thus determines the highest concentrations of lipid uptake which could be detected in co-cultured adipocytes or hepatocytes. This ratio was defined as optimal in our experiments for 10–20 μM of oleic acid loaded onto reconstituted intestinal barriers [11] and the sensitivity of adipocytes with small lipid droplets, i.e. taken at the begining of adipogenesis (5–10 days post) differentiation.

Moreover, lipid droplet sizes (i.e. lipid accumulation) and their number (i.e. differentiation state from multilocular cells to unilocular adipocytes) are under different regulations. Indeed, lipid droplet fusion and growth are under the control of CIDE family proteins [14]. The analysis of fat deposition in terms of droplet number and size brings an original way of analysis. Indeed glucose exerted little effect on droplet number and seems more efficient to increase the volume of lipid droplets (Figure 1(c)), while insulin was more potent to increase the number of lipid droplets. The presence of OA increased both size and number of lipid droplet with a major effect on droplet volume (Figure 1(d)). These results are in accordance with the high transcriptional induction of CIDE family genes by OA in high glucose media and in a lesser extent by insulin [10].

- TG accumulation in fully differentiated adipocytes (up to D15) is related to a dose-dependent (up to OA 200 µM) reduction of cell index in RTCA experiments due to reduction of cell adhesion force [6]. It corresponds to a regular increase in lipid droplet number and size resulting in an increased total TG content (Figure 1(b)). Low differentiated cells are highly sensitive to fatty acid uptake. *In vitro* adipose cell differentiation remains heterogeneous. However in cultures the dose-dependent effect of oleic acid depends on differentiation state (Figure 1(a)). The conditions of cell culture are also critical for OA uptake. The presence of low or high concentration glucose and insulin influence in a major way the capture of OA (Figure 1(b–d)). The cell index of 3T3L1-MBX cells was reduced in OA dose-dependent manner resulting in an IC50 = 1.5 µM (Figure 2(a)). These results are in agreement with previous studies [10,15]. Although the slopes of cell index in response to increasing concentrations of fatty acids are representative of the lipid accumulation inside adipose cells, it is from our experience a wise precaution to confirm these results by measuring the cell lipid content and the cell number to insure these results. Indeed, modification of cell membrane or cell death will results in a decreased cell index not so different from the one observed in response to intracellular lipid accumulation.

- Not only adipose cells but also hepatocytes can be used to quantify the release of fatty acid by the intestinal barrier. Hepatocytes are less adapted than adipose cells to lipid storage. Instead of decreasing in response to lipid accumulation, their cell index increased (Figure 7). That may be explained firstly by a limited amount of lipid stored and secondly by a different cellular response to lipid accumulation. Indeed hepatocyte keep their shape, they do not become spherical suggesting a cytoskeleton structure and response different from the one of adipocytes. Besides, under our experimental conditions, we did not obtained heavily lipid loaded hepatocytes. That was not the objective of the present study.

Interestingly, although adipocytes are much sensitive sensors than hepatocytes for the study of lipid transfer through intestinal barriers, they were highly sensitive to co-culture media, including glucose and insulin concentrations, although lipid uptake by hepatocytes is independant of both parameters. These results are in accordance with their metabolic regulation of adiposity in either healthy, obese and/or diabetic individuals [16]. Therefore, the present study points out the importance of such parameters to adapt the protocols in order to reproduce *in vitro* the site-specificity of nutrient absorption (for example of glucose and fatty acids), the metabolic timing of post-prandial induced modifications such as glycemia and insulinemia in either health or in case of metabolic syndrome as well as other pathologies such as cancers.

Since the uptake of TG by adipose cells can be monitored using xCELLigence, we used these cells as lipid sensors for the quantification of OA uptake and delivery through a reconstructed intestinal barrier. For that purpose we compared intestinal barriers made of Caco-2 cells alone, which originate from colon cancer, or a co-culture made of Caco-2/HT29-MTX (90:10). Adipose cells responded differently in presence of Caco-2 *versus* co-culture. Indeed, cell index of adipose cell decreased in presence of the co-culture while it remained stable in presence of Caco-2, suggesting that the Caco-2 intestinal barrier released almost no lipid. Another observation is that the presence of the co-culture, without oleic acid load was sufficient to induce lipid deposition within adipose cells (Figure 3(a,b)). The decrease in cell index is well correlated with the increased lipid content in adipose cells (Figure 4(a,b)). We also tested the effects of glucose and insulin on the uptake and release of oleic acid by the intestinal barrier because a meal is not only composed of lipids but also carbohydrates and glucose rises plasma insulin concentration. In the present case, high glucose concentration significantly increased TG accumulation in adipose cells while insulin had no significant effect. The lipid content of intestinal barrier showed an exact mirror image of the adipose cell lipid content (Figure 3(b)). This is also confirmed by the fact that the co-culture intestinal barriers retained more lipids than the Caco-2 cells barrier (Figure 5). To test the importance of lipid release from the intestinal barrier we used GLP2 which is known to stimulate postprandial chylomicron production and release of intestinal triglyceride storage pools [12,17]. GLP2 was more efficient on primed intestinal barrier than in non-primed. In primed intestinal barriers GLP2 decreased lipid content and increased that of  adipose cells compared to untreated control (Figure 6). GLP2 increases chylomicron release through increased glycosylation and activity of FAT/CD36 [18]. Although we did not found significant differences in mRNA transcripts of FAT/CD36, the protein levels were found higher in Caco-2/HT29-MTX barriers after priming with OA [11]. FAT/CD36 is mainly involved in the fatty acid induced transcriptional regulations of major genes involved in TG synthesis and its own transcription rather than direct regulation of fatty acid uptake and Peroxisome-Proliferator Activated Receptors (PPARs) signaling [18–21]. Moreover PPAR signaling and target genes such as FABPs and CIDEC are highly dysregulated in Caco-2 *versus* Caco-2/HT29-MTX barriers [11]. In these intestinal barriers, we previously found that OA induced the gene transcription of fatty acid receptor GPR120, perilipin5, fatty acid bing proteins FABP1 and 4, lipid droplet associated protein CIDEB. Together with the loss of lipid transfer control by GLP2 through Caco-2 intestinal barriers (Figure 6), these results indicate that cancer cells increase their capacity to deliver lipids and thus might favor hyperlipemia. Moreover we found that glucose may greatly modulate cancer cell proliferation like in hepatocellular carcinoma cells through LKB1/NADPH Oxidase signaling [22] and other studies are in progress on several cancer cell models in order to depict the signaling pathways dysregulated according to crosstalk between glucose, insulin and fatty acid signaling in cancer cells (Berger & Géloën, manuscripr in preparation).

The measure of the cell index is not only a descriptive observation to study cell modification. The utilization of specific inhibitors of signaling pathways allows the identification of the regulations involved in metabolism. In a previous study we used such a method to delineate the role of FAT/CD36 in adipocytes as well as the signaling pathways involved in the regulation of hepatocellular carcinoma cell proliferation by glucose [11,22]. Numerous examples exist in the literature on the applications of cell index measurement not only to quantify real-time cell proliferation but also to describe the signaling pathways involved [22].

Of course a co-culture model of intestinal barrier is still far from the *in vivo* intestine, although simplistic it brings the opportunity to understand the basic regulations governing the functional relationships between the different cell types. The present model will be valuable to study lipid absorption through intestinal barriers and their most adjacent tissues, i.e. visceral adipose and liver cells, according to lipid composition of the diet, the composition of the microbiota, the impact of either toxins, nanoparticles or drugs, as well as its dysregulation in intestinal cancer, in fatty liver diseases or in visceral adiposity related to obesity or diabetes. The present study pave the way to develop more complex *in vitro* models. For example it would be of great interest to proceed with human primary cell cultures. The aim of the present study was to develop a simple method in order to use adipocytes as sensors for lipid uptake by reconstituted intestinal barriers. For this purpose we took advantage of homogeneous adipogenesis in 3T3L1-MBX cell line rather than direct application to human primary cells since metabolic parameters influencing lipid accumulation in adipocytes, such as glycemia and insulinemia should be taken into account (Figure 1). In addition to higher heterogeneity in human primary cell adipogenesis *in vitro*, such a co-culture model requires experiment reproducibility on several donors sharing similar metabolic parameters including insulin sensitivity or glucose tolerance, as well as for human primary hepatocytes. In future experiments, co-culture models using human primary cells thus would be highly usefull in order to analyze the possible relationships between lipid absorption performances and metabolic disorders such as hepatic steatosis, as well as cancers, in humans.

## 4. Methods

### 4.1. Cell cultures

Mouse 3T3L1-MBX cell line (subcloned from 3T3L1 fibroblasts to ensure close to 100% differentiation to adipocytes and great response to insulin, Sigma Aldrich) were grown in Dulbecco’s Modified Eagle’s Medium with high glucose (4.5 g/L) containing 10% fetal calf serum (DMEM 10% FCS, PAA Laboratories) added with streptomycin plus penicillin (100 units/mL; Sigma Aldrich). After 3T3L1-MBX have reached confluency, differentiation was induced as previously described [10]. Mouse hepatocyte cell line AML12 (LGC Standards) were grown in 1:1 mixture of DMEM and Ham’s F12 medium with 0.005 mg/mL insulin, 0.005 mg/mL transferrin, 5 ng/mL selenium 40 ng/mL dexamethasone (Sigma Aldrich), antibiotics and 10% FCS during one week after confluency before experiments.

Reconstituted intestinal barriers were grown as Caco-2 cells alone or in co-culture with 10% HT29/MTX cells (LGC Standards) in xCELLigence E96-inserts (ACEA Biosciences), during 18 days post-confluency according to previously described [10].

### 4.2. Treatments and co-cultures

Oleic acid was prepared at 200 µM stock solution in free fatty acid bovine serum albumin (BSA) 10% for 2 h at 42°C before dilution in culture media containing 1% bovine serum albumin (BSA) then filtered before use. Differentiated intestinal barriers (D18), were treated at the apical face during 2 h with either oleic acid or vehicle in low glucose (1 g/L) containing DMEM media, adipocytes and hepatocytes in DMEM with high glucose and FCS 10%. Apical media were replaced before loading onto adipocytes or hepatocytes for co-culture during 24 h. After treatments either inserts or plated cells were fixed in formalin 10% before fluorescence imaging.

### 4.3. Real-time cell analysis

Both 3T3L1-MBX and AML12 cells were seeded at 20 000 cells/cm^2^ in either 96-wells or E-view 96 for monitoring respectively on Cytation 3 or on xCELLigence. This system allows label-free monitoring of changes in cell number, morphology and quality of cell attachment measurement on integrated microelectronic sensor arrays. Realtime cell analysis (RTCA) system measures cell surface occupancy, i.e. cell index, taking into account cell number, cell size and adhesion force. Data are represented as delta cell indexes, i.e. differences of cell index *versus* time of treatment, according to time.

### 4.4. Triglycerides storage, cell size and cell survival analyses

TG accumulation was monitored using AdipoRed Assay reagent (Lonza France SARL). Cell number counting was performed using nuclei labeling with Hoechst 33258 (Sigma-Aldrich). Cells were washed in PBS then treated with 0.1% Triton and labeled with Hoechst 33258, 1 mG/mL (Sigma Aldrich). Phase contrast and fluorescent images were obtained with X4 or X20 objectives on Cytation 3 plateform (Biotek Instrument Inc.). Cell culture analyses were performed at magnification x4 as described previously [10]. Briefly, lipid droplets were counted using automated analysis of images acquired with identical parameters with Gen5 2.08 software from Cytation 3. Intracellular accumulation of TG in intestinal barriers, hepatocytes, or in short-term experiments for adipocytes, was expressed in term of fluorescence intensity (CFU) of AdipoRed normalized to Hoechst 33258 (after correction to blank). In adipocytes, TG accumulation was expressed in term of droplet number, i.e. AdipoRed counts (diameter 1–200 µm) normalized to Hoechst 33258 counts (5–50 µm), droplet volumes and full TG content per cell corresponding to (droplet number per cell x mean droplet volume).

Cell size and AdipoRed positive cells were analyzed using AdipoRed labeling of dissociated cells by trypsin 0.05% then fixed with formalin 10% in phosphate buffer saline (PBS) was measured by cytometry on a Novocyte plateform (ACEA, Ozyme) or on a cell size fractionation analyzer (Multisizer, Beckman Coulter).

### 4.5. Data analyses and statistics

Data presented are representative experiments performed at least in triplicates, as mean values ± SEM for RTCA, Cytation 3 counting (n = 8 for 96- and E96-wells plates and 96 wells inserts) and of 8 cumulated wells for cytometry or mulitisizer cell size fractionation analyses. Statistical analysis were performed with Stat View 4.5 software for Windows, the data were analyzed using Student’s t-test and one way ANOVA followed by Fisher’s protected least significance difference [PLSD], *post hoc* test. Significance level was accepted at p < 0.05.
